# Alterations in the Properties of Red Blood Cells in Men with Coronary Artery Diseases after Comprehensive Cardiac Rehabilitation

**DOI:** 10.1155/2020/6478785

**Published:** 2020-11-26

**Authors:** Krzysztof Gwozdzinski, Anna Pieniazek, Joanna Bernasinska-Slomczewska, Joanna Brzeszczynska, Robert Irzmanski, Anna Jegier

**Affiliations:** ^1^Department of Molecular Biophysics, Faculty of Biology and Environmental Protection, University of Lodz, Lodz 90–236, Poland; ^2^Department of Internal Medicine and Cardiac Rehabilitation, Medical University of Lodz, Lodz 90–647, Poland; ^3^Department of Sports Medicine, Medical University of Lodz, Lodz 92–213, Poland

## Abstract

**Purpose:**

Comprehensive cardiac rehabilitation (CCR) is a complex program aimed at improving the health status of patients with coronary artery disease (CAD), especially those who have been subjected to cardiac interventions (PCI and CABG).The aim of this study was to measure the changes in the properties of red blood cells (RBCs) in men with CAD after cardiac intervention and after participation in CCR program.

**Methods:**

In this study, we have investigated the influence of the physical training-based CCR program in 12 men with CAD, after PCI or CABG. The characteristics of RBCs including the basic morphology of RBCs, the conformational state of RBC membrane protein and hemoglobin, acetylcholinesterase activity, membrane fluidity, the osmotic fragility, and thiol concentration in membrane and in hemolysate were measured. Ascorbate concentration and reduced glutathione were also determined. The analysis was performed in men, before and after participation in CCR. The properties of RBCs were observed in connection with the exercise test, and parameters were evaluated before, immediately after, and 1 hour after the exercise test.

**Results:**

After CCR, a decrease in the mobility of erythrocyte membrane proteins was observed, which was accompanied by a decrease in lipid fluidity. In addition, immediately after the exercise test and 1 hour later, we measured a decrease in thiol level in hemolysate, but not in the plasma membrane. Unexpectedly, an increase in reduced glutathione concentration one hour after the exercise test after completing comprehensive cardiac rehabilitation was observed.

**Conclusion:**

CCR in men with CAD after cardiac intervention is connected with decreased membrane fluidity and decreased membrane protein mobility, which indicates that reduction of oxidative changes in these components occurs.

## 1. Introduction

Cardiac interventions such as percutaneous coronary intervention (PCI) and coronary artery bypass grafting (CABG) are employed as the main method of treatment in cardiac patients with coronary artery disease (CAD) [1]. Both, preexisting (CAD) and performance of cardiological interventions (e.g., CABG), are linked to the inflammation and oxidative stress development [2, 3].

Physical activity has been shown to influence the patient's fitness, morbidity, and mortality. It has been proven that acute exercise may induce oxidative stress and symptoms of their disease in cardiopulmonary patients [4]. Exercise has also been found to trigger an increase in lipid peroxidation of blood plasma in patients with chronic heart failure (CHF) [5]. Nevertheless, mild intensity of physical activity has been demonstrated to positively affect some of the risk factors responsible for the development of CAD, such as lowering hypertension in patients [6]. Repetitive physical activity of mild intensity may have beneficial effect on cardiac patients, even after acute ischemic cardiac events [7]. There is growing evidence that patients who have undergone cardiac interventions such as PCI and CABG may have favorable health effects if they engage in comprehensive cardiac rehabilitation (CCR) through mild physical exercise, even in the early stages of recovery [8]. Successful rehabilitation of CAD patients must involve education regarding risk factors, good nutrition, and employment of dietary changes and supplementation [9, 10]. Moreover, control of blood glucose level and lipids [11], and control of blood pressure [12] with the introduction of physical training are one of the crucial factors of comprehensive CCR program [13].

Physical training, as a part of CCR programs, has been shown to postpone the progression of the disease, resulting in an improvement of the patient's physical state, thereby allowing the patient to perform normal daily living activities. Physical training, included in both CCR and a root for secondary prevention, has been shown to lower the risk of repetitive cardiovascular events and has been shown to increase survival rates [14]. Overall, modern comprehensive CCR is a safe and proven tool for the management of CAD patients [13].

Moderate physical training, appropriately matched with the fitness level of cardiac patients, leads to significantly increased exercise capacity through induction of multiple physiological changes in the body. These alterations include changes in blood biochemistry and hematology, hormonal status, and metabolism [15]. Physical training increases blood oxygenation, tissue perfusion, and oxygen metabolism, which can lead to mild oxidative stress. Exercise-induced mild oxidative stress can further evoke adaptive changes, such as increased expression of antioxidant enzymes causing a general improvement in the function of tissue antioxidative systems. Of note, exercise-based CCR has also been proven to reduce the level of oxidative stress [16].

Although physical training-induced alterations in the blood influence changes in other tissues, oxidative stress-related effects of exercise on blood have not been extensively investigated and are not entirely understood [17]. Therefore, this study was designed to investigate the influence of the comprehensive physical training-based CCR program on blood parameters.

Changes to the properties of RBCs can lead to impaired oxygen supply to other cells and tissues resulting in hypoxia. Our previous work presented significant changes in the plasma and red blood cell parameters during exercise [17, 18].

This study aims to examine whether cardiological rehabilitation can cause beneficial changes in the structure and function of red blood cells and in selected plasma parameters. We discussed the influence of comprehensive cardiac rehabilitation on red blood cells and other obtained changes at the cellular level and their contribution to CCR-related outcomes. Selected plasma parameters and red blood cell properties were examined in patients' blood before and after the exercise tests.

## 2. Material and Methods

### 2.1. Chemicals

4-Maleimido-2,2, 6,6,-tetramethylpiperidine-1-oxyl (MSL), 4-iodoacetamide-2, 2, 6, 6, -tetramethylpiperidine-1-oxyl (ISL), 12-doxyl-stearic acid (12-DS), o-phthalaldehyde (OPA), N-ethylmaleimide (NEM), quinaldic acid, pyridine, 4, 4′-dithiodipyridine (DTDP), and 5, 5′-dithio-bis (2-nitrobenzoic acid) (DTNB) were obtained from Sigma Chemical Co. (St. Louis, MO). All other chemicals were of analytical grade procured from POCh (Gliwice, Poland).

### 2.2. Subjects and Experimental Procedures

Cardiac patients underwent interventions including percutaneous coronary intervention (PCI) and coronary artery baypass graft (CABG). The cohort of 12 male volunteers, aged 52.2 ± 6.4 years, weighing 84.2 ± 12.9 kg, with a BMI of 29.4 ± 4.5 was examined before and after physical training. The physical exertion was based on comprehensive cardiac rehabilitation (CCR). The mean time since medical intervention started was 22 ± 9 days in the PCI group and 28 ± 9 days in the CABG group. The study was conducted in the Outpatient Cardiac Rehabilitation Center of Clinical University Hospital. All the patients evaluated were prescribed the following medications: acetylsalicylic acid, angiotensin-converting-enzyme inhibitor, statin, and *β*-blocker. Only the patients who fully completed CR program and did not require modification in pharmacotherapy during program were included in the study. The outpatient CCR program was composed of 24 physical training bouts and a single education session on cardiovascular risk factors and lifestyle modification including the recommendations concerning healthy diet, psychological support in stress management, and effective methods of fighting addictions, including nicotine.

The participants performed interval physical training, with continuously monitored 12-lead electrocardiography and blood pressure recordings, for 35 minutes 3 times a week on cycle ergometers Ergoselect II 100/200 Ergoline Reha System GmbH, Schiller, Switzerland. Training sessions were conducted with increasing workloads in the first part and decreased in the second part, alternating between 4 minutes of workload and 2 minutes of active restitution. The peak training intensity in both examined groups was determined by training heart rates (THRs), calculated during the exercise test, from heart rate reserve (HRR) according to the Karvonen formula [19]:

(1) $$\begin{array}{c} \text{THR}={\text{HR}}_{\text{rest}}+\left(0.6\text{ to }0.8\right)\times \text{HRR.} \end{array}$$

Heart rate reserve (HRR) was calculated as the difference between the highest heart rate achieved during the exercise test (HR peak) and resting HR (HR rest). The intensity of training was adjusted so that the patient's heart rate achieved the calculated THR but did not exceed this value. Training intensity ranged from 11 to 13 points on the Borg scale [20] in the initial training sessions and from 14 to 16 points on the Borg scale in the advanced stages of training.

A symptom-limited, multistage exercise test was performed in every patient using cycle ergometer Ergoselect II 100/200 with 12-lead continuous electrocardiography monitoring with Cardiovit CS-200 ErgoSpiro, Schiller, Switzerland. At the beginning of the exercise test, the workload was set at 60 W and was gradually increased by 30 W every three minutes until exhaustion. The criteria for the exercise test termination included the occurrence of clinical symptoms such as angina, dizziness, headache or breathing problems, abnormal ECG findings, blood pressure values above 250/115 mmHg, exhaustion, or the individual's inability to maintain the pedaling frequency of 60 revolutions/min. After exercise completion, the participants were monitored for approximately six minutes. A cooldown phase consisted of 2 minutes of unloaded pedaling.

Following the exercise, the subjects remained seated at rest for 1 h and only water intake was allowed during that time. Venous blood samples were drawn from the antecubital veins of the patients two times: at the beginning of CCR and at the end of it. Each time, the samples were collected before the exercise test, immediately after exercise, and 1 h after the exercise session. Morphological parameters of blood in patients with CAD before and after CCR were determined using an autoanalyzer.

The experiments were performed in accordance with the guidelines of the Declaration of Helsinki and conformed to the ethical principles set by the Belmont Report, Ethical Principles and Guidelines for the Protection of Human Subjects of Research. All the participants signed an informed consent form prior to their enrolment into the study.

### 2.3. Preparation of Erythrocytes, Hemolysate, and Erythrocyte Membranes

For experiments, blood was centrifuged at 4°C and the erythrocytes were washed thrice using 10 mmol/L phosphate buffer saline having a pH of 7.4 (PBS). Packed cells were suspended in PBS to a hematocrit of 50%.

The hemolysate was obtained from washed RBCs by the addition of 10 mmol/L phosphate buffer with a pH 7.4 (volume ratio 1 : 1). The hemolysate was centrifuged at 4000 × g for the elimination of erythrocyte ghosts. The hemoglobin concentration in the hemolysate was measured by Drabkin's method [21].

The erythrocyte plasma membranes were isolated by washing the cells several times with the decreasing ionic strength of phosphate buffer at pH 7.4 (from 20 mmol/L to 5 mmol/L) and 4°C, according to the modified method of Dodge [22]. Protein concentration was evaluated using Folin reagent by the method of Lowry et al. [23].

### 2.4. Spin Labeling of Hemolysate and Plasma Membrane Proteins

In order to investigate the conformational changes of hemolysate and plasma membrane proteins, MSL and ISL spin labels were applied. Hemolysate and plasma membrane proteins were labeled using MSL or ISL (50 : 1). In order to remove the unbound spin labels in hemolysate, 24 h of dialysis against 10 mmol/L phosphate buffer was executed. Unbound spin markers in the cell membrane proteins were then removed by repeated washing in 5 mmol/L phosphate buffer at pH 7.4.

The EPR spectra of MSL and ISL attached to the plasma membrane proteins or hemolysate proteins were used to calculate the *h*_+1_/*h*_0_ ratio, where *h*_+1_ and *h*_0_ represent the EPR high-field and center lines, respectively.

### 2.5. Plasma Membrane Fluidity

Lipid membrane fluidity of erythrocytes was estimated using 12-DS spin label by EPR technique. The spin label (0.1 mmol/L) in ethanol solution was added to the erythrocytes (hematocrit 50%). The EPR spectra of 12-DS, incorporated to erythrocyte membrane, were used to calculate the *h*_–1_/*h*_0_ ratio, where *h*_–1_ and *h*_0_ represent the EPR low-field and center lines, respectively.

EPR spectra were recorded on a Bruker ESP 300 ESR spectrometer at room temperature (21 ± 2C). The internal settings of the instrument included a microwave frequency of 9.73 GHz, a microwave power of 10 mW, the center field set at 3480 G with a range of 80 G, a modulation frequency of 100 kHz, and a modulation amplitude of 1.01 G.

### 2.6. Thiol Measurements

The concentration of the thiol groups in the hemolysate was measured using the method described by Egwim and Gruber [24]. Thiols react with 4, 4′-dithiodipyridine to form 2-thiopyridon, which show absorbance at 324 nm.

The plasma membrane thiol groups were measured using the method described by Ellman [25]. The thiols reacted with DTNB to form anions with a strong yellow color, which show absorbance at 412 nm.

For both the methods, calibration curves were prepared using different concentrations of GSH and calculated as nmol/mg of protein or Hb.

### 2.7. Acetylcholinesterase Activity

The membrane acetylcholinesterase activity was measured spectrophotometrically using Ellman's method [26]. An estimation of absorbance slope was performed for 1 min at 412 nm. The rate of reaction of hydrolysis of acetylthiocholine iodide was calculated as follows:

(2) $$\begin{array}{c} V=\frac{\Delta A\times F}{13600\times 1000}, \end{array}$$

where Δ*A* is the increase in the absorbance in 1 min, F is the dilution factor, and 13600 mol L^−1^ × *l* × cm^−1^ is the extinction coefficient for 5-thio-2-nitrobenzoic acid. Acetylcholinesterase activity was expressed as *μ*mol of acetylthiocholine that was degraded in 1 min by acetylcholinesterase contained in 1 mg protein.

### 2.8. Ascorbic Acid

The ability of ascorbic acid to reduce iron (III), as described by Arya and Mahajan, was used to measure ascorbic acid [27]. Iron (II) thus formed is complexed with quinaldic acid and pyridine to form a colored compound, which shows absorbance at 380 nm. The concentration of ascorbate in the sample was estimated from the standard curve prepared for different concentrations of ascorbic acid (in the range of 0–0.2 mmol/L) and calculated as nmol/mg of Hb.

### 2.9. Reduced Glutathione

The reduced glutathione (GSH) concentration was determined using o-phthalaldehyde (OPA) by the fluorimetric method [28]. The reaction product of OPA and GSH has a high fluorescence quantum yield. The OPA-derived fluorescence was measured at an excitation wavelength of 365 nm and emission at 430 nm. The glutathione concentration was calculated using the calibration curve for different concentrations of reduced glutathione and expressed as nmol/mg hemoglobin.

### 2.10. Erythrocyte Osmotic Fragility

The osmotic fragility of erythrocytes was determined by the method described by Morimoto et al. [29]. The erythrocytes (50% hematocrit) were suspended in solutions containing different concentrations of NaCl (0–155 mmol/L). The samples were centrifuged and absorbance of the supernatant was measured at 540 nm. The osmotic fragility were presented as the NaCl concentration, for which 50% of the erythrocytes underwent hemolysis.

### 2.11. Statistical Analysis

The results were expressed as a mean ± standard deviation. The normality of data was tested using the Shapiro–Wilk test while variance homogeneity was verified using the Brown–Forsythe test.

The statistical significance between three different time periods of the withdrawal of blood during exercise as well as before and after CCR was estimated using analysis of variance by repeated measures. Each difference was estimated using the test for planned comparisons. All statistical analyses were conducted in STATISTICA.PL v.13.0.

## 3. Results

The investigated parameters that were associated with oxidative stress were monitored in whole RBCs, in hemolysate and isolated plasma membranes. In addition, the physiological parameters of RBCs in blood were obtained from patients with CAD before and after CCR were evaluated. A significant increase in the level of hemoglobin, hematocrit, and the number of erythrocytes in blood was found in the samples obtained immediately after physical exercise, in comparison to those obtained prior to the exercise before CCR. After recovery (1 h), these parameters were decreased to a level comparable to the preexercise values (Table 1). There were no differences in the corpuscular volume of erythrocytes and corpuscular volume of blood hemoglobin in patients with CAD. Furthermore, similar results were also observed after CR (Table 1).

**Figure 1:**
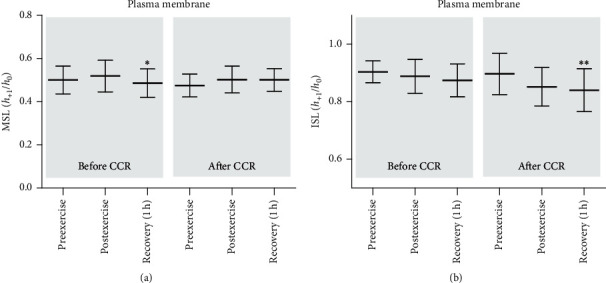
Alterations in *h*_+1_/*h*_0_ ratio of MSL and ISL spin labels attached to the plasma membrane protein in the patients with coronary artery disease before and after comprehensive cardiac rehabilitation. Significant differences: ^*∗*^before CCR, postexercise vs. recovery (1 h at *p* < 0.05). ^*∗∗*^After CCR, preexercise vs. recovery (1 h *p* < 0.05).

In order to investigate the conformational state of hemoglobin in the hemolysate and protein in plasma membrane, the electron paramagnetic technique was employed. The maleimide spin label covalently attached to the membrane proteins showed significantly lower mobility in the samples taken from CAD patients postexercise before the rehabilitation, in comparison to the samples taken after 1 h of recovery (Figure 1(a)). In samples taken after the end of CCR, no such differences were observed. However, after the completion of CCR, an decrease in the mobility of the iodoacetamide spin label attached to plasma membrane proteins was observed after 1 h of recovery in comparison to the preexercise values (Figure 1(b)). The mobility of either of the spin labels (MSL and ISL) attached to hemoglobin in hemolysate did not show a significant change either before CCR or after the termination of rehabilitation (Table 2).

**Figure 2:**
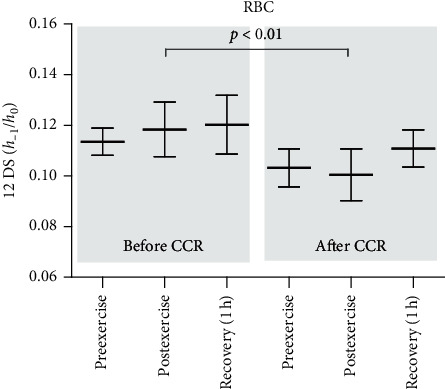
Changes in in *h*_−1_/*h*_0_ ratio of 12-DS incorporated to whole erythrocytes of patients with coronary artery disease before and after comprehensive cardiac rehabilitation. ^*∗*^Significant difference between before CCR (postexercise) and after CCR (postexercise) at *p* < 0.01.

EPR was also used for the determination of fluidity of erythrocyte plasma membrane. A significant decrease in the *h*_–1_/*h*_0_ parameter of RBCs was observed after CCR (postexercise) in comparison to that measured before CCR (postexercise) (Figure 2). On the other hand, no differences were observed in the osmotic fragility and acetylcholinesterase activity in the erythrocytes before and after CCR (Table 2).

**Figure 3:**
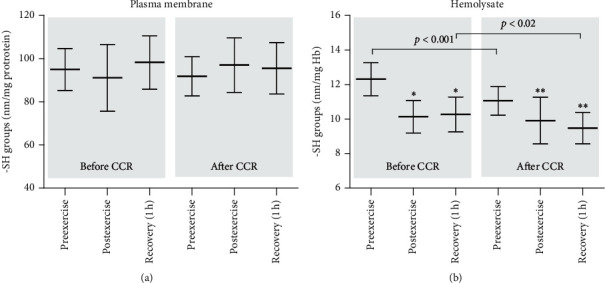
Thiol group concentration in the plasma membrane (a) and hemolysate (b) proteins in patients with coronary artery disease before and after comprehensive cardiac rehabilitation. Significant differences: ^*∗*^before CCR, preexercise vs. postexercise and recovery (1 h at *p* < 0.005). ^*∗∗*^After CCR, preexercise vs. postexercise and recovery (1 h at *p* < 0.005).

The results obtained did not show any difference in the concentration of thiol groups in the erythrocyte membrane proteins either before CCR or after the termination of rehabilitation (Figure 3(a)). Contrarily, alterations in the level of thiol groups were observed in the hemolysate proteins in the samples obtained before and after CCR and during physical activity. It was identified that both, before and after the rehabilitation, the levels of thiol groups in proteins decreased significantly in the hemolysate in the postexercise samples and after 1 h of recovery in comparison to the preexercise samples (Figure 3(b)). In addition, it has been demonstrated that CCR causes a significant decrease in the level of thiol groups in proteins in hemolysate (Figure 3(b)).

**Figure 4:**
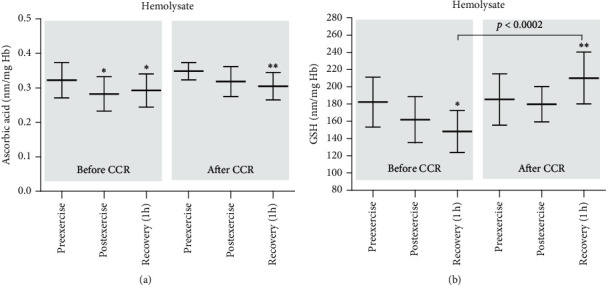
Concentration of ascorbic acid (a) and reduced glutathione (b) in the hemolysate from patients with coronary artery disease before and after comprehensive cardiac rehabilitation. Significant differences: ^*∗*^before CCR, preexercise vs. postexercise and recovery (1 h at *p* < 0.005). ^*∗∗*^After CCR, preexercise vs. recovery (1 h at *p* < 0.005).

Ascorbic acid and reduced glutathione are the most abundant antioxidants present in cells. The results obtained in the present study show that the concentration of ascorbic acid in hemolysate dropped significantly (before CCR) after exercise and after 1 h of recovery in comparison to that before exercise (Figure 4(a)). After CCR, a significant decrease in ascorbic acid concentration was observed just after 1 h of recovery in comparison to the preexercise values (Figure 4(a)). On the other hand, before CCR, a significant decrease in glutathione concentration in samples taken after 1 h of recovery was observed in comparison to the preexercise level (Figure 4(b)). Unexpectedly, after CCR, the concentration of glutathione after 1 h of recovery was significantly higher in comparison to the preexercise levels (Figure 4(b)). In either case of the antioxidants, the initial levels before and after CCR did not change.

## 4. Discussion

The spectrum of IHD includes stable angina, unstable angina, myocardial infarction, and sudden cardiac death. IHD is associated with a decreased blood flow to the heart muscle, due to narrowing of the coronary artery resulting from the development of atherosclerotic plaque. The decrease in blood perfusion creates hypoxia in the heart muscle. Oxygen demand is crucial for cardiac function and survival, as well as for the regulation of gene expression, which maintains fenotype and functions of cardiomyocytes [30]. Oxygen is also important for the release of nitric oxide, which plays a key role in the regulation of vascular tone, cardiac contractility. Additionally, hypoxia also leads to decreased antioxidant defense in the heart muscles and cardiomyocytes. The decreased activity of superoxide dismutase (SOD) and glutathione has been observed to occur under hypoxic conditions [31]. Oxygen is also necessary for the generation of ROS, which are important molecules in the cell signaling process, nevertheless they also exert detrimental effects that contribute to cardiac dysfunction and death [32]. CCR aims to prevent heart disease, increasing the patient's quality of life, along with limiting the progress of CAD and reducing mortality [33].

Changes in the RBCs and their components were examined before, immediately after, and one hour after exercise. RBC parameters in CAD patients before and after CCR were measured. Significant increase in the hematocrit, the number of RBCs, and hemoglobin was observed immediately after physical exercise when compared to levels before exercise, both prior to and after CCR. Immediately after exercise before and after CCR, all three parameters showed an increase of approximately 5%. After one hour of recovery, all parameters returned to levels comparable to those before exercise. Statistically significant increase in these parameters was also reported in a group of young untrained men after exercise [34]. The increase in hemoglobin and hematocrit in the obtained samples, immediately after exercise, could be due to plasma volume changes following exercise. However, an insignificant decrease in hematocrit, the number of erythrocytes, and hemoglobin was observed after CCR. In contrast, an increase in hematocrit and the number of erythrocytes was reported after CCR in a previous study [35].

Cell membrane studies are important because its fluidity determines the deformability of the RBC in the microcirculation. Changes in liquidity also reflect the function of the membrane system, necessary for cell function and disposal of toxic metabolites. It has been shown that oxidative damage to erythrocyte membrane results in the deterioration of its deformability and impaired oxygen transfer [36]. It was demonstrated that oxidative damage of the erythrocyte membrane has effect on its viscoelastic properties [37]. Additionally, oxidative stress has been shown to lead to echinocyte formation [38].

ROS can oxidize proteins, enzymes, lipids, and other macromolecules. In addition to the oxidation of amino acid side chains, oxidation of peptide backbones, protein-protein cross-linking, and advanced oxidation protein products (AOPPs), carbonyl compounds are also released, and decrease in the thiol group concentration in plasma was observed [39].

Lipid and protein properties in the plasma membrane were measured using the spin labeling method in EPR spectroscopy. The conformational state of membrane proteins, as well as hemoglobin in hemolysate, was determined using two covalently spin labels, MSL and ISL. Decrease of *h*_+1_/*h*_0_ ratio reflects a decrease in the mobility of MSL attached to the membrane proteins was observed postexercise in comparison to that after 1 h recovery before CCR. These results reflect the alterations in the conformation of membrane-cytoskeleton of the erythrocyte membrane. It has previously been shown that 75–90% of MSL are bound to spectrin-actin cytoskeletal complex [40]. Similarly, in the case of ISL, a decrease in the mobility of this label after 1 h recovery was found in comparison to preexercise value. In contrast to MSL, this label connects to peripheral proteins. For examination of lipid membrane fluidity, the spin-labeled fatty acid 12-DS was used. The reporter group of this label is located at the depth of the lipid monolayer, which is rich in double bonds. Using 12-DS, a decrease in lipid membrane fluidity at 1 h postexercise before rehabilitation compared to the same point after CCR was demonstrated in this study. A decrease in the *h*_−1_/*h*_0_ ratio of spin-labeled fatty acids in the erythrocytes was also observed in a group of young untrained men, one hour after exhaustive exercise [18]. Additionally, it was found that the osmotic fragility of RBCs after CCR (1 h postexercise) showed a tendency to decrease, as compared to that before CCR, which shows a decrease in the sensitivity of these cells to osmotic stress. Proteins are more sensitive to oxidation by ROS than lipids, though the oxidation of both proteins and lipids consequently leads to impaired membrane permeability and alterations in metabolic processes within the cells. The present study revealed positive structural alterations in the plasma membrane of RBCs. Nonetheless, the structural alterations were not a consequence of lipid and protein oxidation. It was shown that lipid and protein oxidation in erythrocyte membrane leads to an increase in their mobility [41]. The results of our study are consistent with those of Taty Zau et al. [42], which demonstrate a decrease in thiobarbituric acid reactive substances (TBARSs) and carbonyl levels after CCR. It seems that changes in lipid fluidity are rather associated with alterations in protein-lipid interaction. In addition, no changes were observed either in the conformational state of hemoglobin in hemolysate for the spin labels attached to this protein or in acetylcholinesterase activity.

Thiol groups affect the regulation and maintenance of intracellular redox status in red blood cells. Human red cells rich in SH groups present in enzymes and proteins are an easy target for ROS [39]. Thiols, such as glutathione and other low-molecular-weight thiols, play an important role in the protection of cells and tissues from oxidative stress. No difference in the level of thiols in plasma membranes of erythrocytes was observed before and after CCR during the examined time periods (before, immediately after exercise, and 1 h after the end of the exercise). This is further evidence that the processes associated with oxidation of proteins and lipids do not occur in the erythrocyte membrane. In contrast, a significant decrease in thiols was observed in hemolysate before CCR, immediately after exercise, and an hour later. Slightly similar results were also observed after CCR, although the differences were not statistically significant. Not significant decrease in the thiol levels after exercise and an hour later was also evident in a group of young untrained men [34]. The glutathione levels showed similar tendencies like those of the thiols. Before CCR, just after the exercise, statistically not significant decrease in the thiol level was observed; however, after 1 h of recovery, the decrease in glutathione was significantly greater (approx. 25%) in comparison to the concentration before the exercise. Undoubtedly, an interesting result was the unchanged level of GSH after the exercise and its statistically significant higher concentration one hour after recovery in the group of people after CCR. The analysis of another low-molecular-weight antioxidant, ascorbic acid before CCR, showed a decrease in its concentration immediately after exercise and one hour after recovery. However, a milder decrease in its concentration was observed after CCR. These results are comparable to those of our previous study. The decrease in the concentration of ascorbic acid just after the end of the exercise and an hour later was observed in the group of young untrained men [34].

Generally, CCR attenuates oxidative stress in CAD patients [42]. It has been established in this study that the rehabilitation training led to a reduction in oxidative stress indicators such as malonyldialdehyde and carbonyl compounds, as well as an increase in total antioxidant capacity including glutathione in patients after CCR [42]. Additionally, an increase in the enzyme activities of antioxidants such as SOD-1, GSH-Px, and catalase was demonstrated [42, 43].

## 5. Conclusion

The observed changes in the structure of the erythrocyte cell membrane after CR, e.g., the decrease in lipid membrane fluidity and decrease of the motion of membrane protein cytoskeleton, lead to improvement of rheological properties of RBCs. Changes in the plasma membrane improve oxygen supply to cells and tissues.

## Figures and Tables

**Table 1:** Morphological parameters of blood of patients with coronary artery disease before and after comprehensive cardiac rehabilitation.

Parameter	Before CCR			After CCR		
	Preexercise	Postexercise	Recovery (1 h)	Preexercise	Postexercise	Recovery (1 h)
HGB (g/dL)	14.2 ± 1.1	14.9 ± 1.1^*∗*^	14.3 ± 1.1^#^	13.9 ± 1.6	14.5 ± 1.6^*∗∗*^	13.7 ± 1.7^##^
RBC × 10^6^/*µ*L	4.66 ± 0.4	4.87 ± 0.4^*∗*^	4.71 ± 0.4^#^	4.55 ± 0.6	4.76 ± 0.5^*∗∗*^	4.51 ± 0.5^##^
HTC (%)	42.9 ± 2.6	44.9 ± 2.7^*∗*^	43.3 ± 2.3^#^	41.2 ± 4.8	43.2 ± 4.6^*∗∗*^	40.9 ± 4.9^##^
MCV (fL)	92.5 ± 3.6	92.6 ± 3.7	92.2 ± 3.8	90.8 ± 3.8	91.1 ± 3.9	90.7 ± 3.9
MCH (pg)	30.5 ± 1.6	30.6 ± 2.0	30.3 ± 1.6	30.5 ± 1.9	30.6 ± 1.9	30.3 ± 1.7

HGB: hemoglobin concentration; RBC: red blood cell; HTC: hematocrit; MCV: mean corpuscular volume; MCH: mean corpuscular hemoglobin. Significant differences: ^*∗*^*p* < 0.005 preexercise vs. postexercise (before CCR). ^#^*p* < 0.005 postexercise vs. recovery (1 h) (before CCR).

**Table 2:** Changes in the properties of hemoglobin in hemolysate and protein in membranes of erythrocytes in patients with coronary artery disease before and after CCR.

Parameter		Before CCR			After CCR			*p*
		Preexercise	Postexercise	Recovery (1 h)	Preexercise	Postexercise	Recovery (1 h)	
Hemoglobin in hemolysate	MSL (*h*_+1_/*h*_0_)	0.82 ± 0.06	0.84 ± 0.04	0.87 ± 0.05	0.80 ± 0.10	0.85 ± 0.09	0.85 ± 0.06	n.s.
	ISL (*h*_+1_/*h*_0_)	0.69 ± 0.12	0.70 ± 0.10	0.71 ± 0.09	0.72 ± 0.10	0.73 ± 0.07	0.74 ± 0.06	n.s.
Plasma membrane	AChE *µ*mol/min × mg protein	1.20 ± 0.15	1.14 ± 0.23	1.18 ± 0.21	1.19 ± 0.15	1.17 ± 0.22	1.16 ± 0.16	n.s.
RBC	C_(50)mmol/L_	79.3 ± 3.9	79.6 ± 3.6	79.2 ± 4.1	78.7 ± 7.1	78.6 ± 4.0	77.2 ± 4.4	n.s.

## Data Availability

The datasets used and/or analysed during the current study are available from the corresponding author on reasonable request.

## Abbreviations

PCI: Percutaneous coronary intervention
CABG: Coronary artery bypass grafting
CVD: Cardiovascular diseases
CAD: Coronary artery disease
IHD: Ischemic heart disease
MI: Myocardial infarction
CCR: Comprehensive cardiac rehabilitation
EPR: Electron paramagnetic resonance.
